# Auxiliary two-staged partial resection liver transplantation

**DOI:** 10.1186/s12893-021-01265-5

**Published:** 2021-05-28

**Authors:** Pål-Dag Line, Silvio Nadalin, Deniz Balci

**Affiliations:** 1 Department of Transplantation Medicine, Oslo University Hospital, Nydalen, Postboks 4950, 0424 Oslo, Norway; 2 Institute of Clinical Medicine, University of Oslo, Oslo, Norway; 3 Department of General, Visceral and Transplant Surgery, University Hospital Tübingen, Tübingen, Germany; 4 Department of Surgery and Liver Transplantation Unit, Ankara University School of Medicine, Ankara, Turkey

## Abstract

A case report of two patients who underwent auxiliary liver transplantation and two staged hepatectomy was recently published in BMC Surgery. The surgical technique utilised is described as novel but has been published previously also in the setting of chronic liver disease. A new name for this surgical approach therefore seems redundant. The importance of careful hemodynamic monitoring of pressure and flow in the portal vein and artery of the auxiliary graft as well as optimizing venous outflow is paramount to ensure graft regeneration and avoid small for size syndrome. The relevant surgical considerations to ensure optimal safety has also been reported in previous literature. This brief letter to the editor of BMC Surgery gives an overview that put the article content in context with published literature on this transplant surgical technique.

## Background

We have read the case report from S. Brunner et al. in the recent issue of BMC Surgery [1] and believe that a critical appraisal of the article content in relation to already published literature could be of value for the readers of BMC Surgery.

Progress and improvement in clinical medicine is reliant on original and novel scientific contributions as well as stepwise improvements of existing published treatments and concepts. Brunner and co-workers choose to term the described surgical technique as “a new treatment concept for patients with end-stage liver disease» and propose a dedicated acronym for it. We believe this to be incorrect and contributing to confusion in the terminology of the HPB surgical literature [2].

The technique of combining liver resection with auxiliary liver transplantation has been known since 2015 as the RAPID technique (Resection and partial liver transplantation with delayed hepatectomy) in deceased and living donation respectively [3, 3]. Utilising small liver grafts raises two fundamental problems to be solved: maintaining a sufficient liver metabolic mass and avoidance of the small-for-size syndrome with portal hyperperfusion and resultant arterial vasoconstriction. The technical description devised in the present article is not in any substantial way different from the RAPID concept except from the fact that the authors have used larger grafts (left lobe as opposed to left lateral segments) and that the recipients had end-stage liver disease with portal hypertension instead of malignant liver tumours.

The original RAPID protocol includes the relevant portal modulation procedures, and successful performance of the RAPID technique in patients with cirrhosis through careful modulation of portal vein perfusion has been published previously [5]. In the cirrhotic setting, the portal flow and pressure measurements are critically important to prevent small-for-size situation both after the 1st and 2nd stage since portal hyperperfusion may occur also after the native liver is out. Volume flow measurements of both the portal and arterial circulation is essential in both stages of this procedure, and duplex ultrasound is insufficient in assessing volume flow. The deportalisation in the cirrhotic setting enables accurate assessment of the inflow dynamics and decision making for further modulation that could be planned real-time during the operation [6].

Selective attention to the published literature and introduction of established surgical techniques as novel concepts may limit the collective stepwise improvement of surgical techniques. The authors experienced a range of serious complications that certainly are of great concern, and suggest some modifications based on an “optimized technical approach”. The main learning experiences stated in the current paper are already described in other publications such as end to side anastomosis of the graft portal vein to the main portal trunk, the need to resect segments 1–3 as opposed to 2–3, risk of venous outflow obstruction with increasing graft regeneration necessitating endovascular stenting [3, 7]. Modulation of portal inflow in the setting of small grafts either through splenic artery ligation, splenectomy, or hemi-portocaval shunting is also well established in the liver transplant literature, including in application of the RAPID technique in cirrhotic livers [5, 6, 8]

No other report on this technique have found it necessary to delay the biliary reconstruction to a separate day, pack the liver graft, or mandate a second-look procedure, thus making the patients undergo three laparotomies in the course of three weeks. In our experience, this is not justified and increases both the risk and the complexity of the procedure.

On this background, we believe that the paper in its current form provides a somewhat skewed presentation of the RAPID technique and therefore think the readers of BMC Surgery should be made aware of this. We cannot see any justification or added value for the proposed acronym “ASPIRE” apart as a designated naming of a prospective trial. There is however no citing of ethical approval or study registration in this paper to suggest that this is the case.

## Data Availability

Not applicable.
